# Supplementary material for: The Systems Biology Research Tool: evolvable open-source software
Source: BMC Syst Biol. 2008 Jun 29;2:55. doi: 10.1186/1752-0509-2-55 (PMC2446383; doi:10.1186/1752-0509-2-55)
Supplement: Additional file 1 — SBRT Archive. An archive of the current version of the Systems Biology Research Tool. [file 1752-0509-2-55-S1.zip › sbrt-1.4.0/doc/developers_guide/api/sbrt/shell/text/fba/class-use/FbaOptFileLineFormat.html]

Uses of Interface sbrt.shell.text.fba.FbaOptFileLineFormat


|  |  |  |
| --- | --- | --- |
| **Overview** \| **Package** \| **Class** \| **Use** \| **Tree** \| **Deprecated** \| **Index** \| **Help** | |  |
| PREV   NEXT | **FRAMES**    **NO FRAMES**     **All Classes** |  |


---


## **Uses of Interface sbrt.shell.text.fba.FbaOptFileLineFormat**

| Packages that use FbaOptFileLineFormat | |
| --- | --- |
| **sbrt.shell.io.fba** | Provides classes and interfaces for reading and writing files relevant to Flux Balance Analysis. |
| **sbrt.shell.text.fba** | Provides classes and interfaces for defining formats for objects relevant to Flux Balance Analysis. |

Uses of FbaOptFileLineFormat in sbrt.shell.io.fba

| Methods in sbrt.shell.io.fba that return FbaOptFileLineFormat | |
| --- | --- |
| `FbaOptFileLineFormat` | `ColumnOrFbaOptOutputFile.getFbaOptFileLineFormat()`             Returns the FBA optimization file line format used to write data strings to this file. |
| `FbaOptFileLineFormat` | `SerFbaOptOutputFile.getFbaOptFileLineFormat()`             Returns the FBA optimization file line format used to supply a header object for objective values. |
| `FbaOptFileLineFormat` | `RowOrFbaOptOutputFile.getFbaOptFileLineFormat()`             Returns the FBA optimization file line format used to write data strings to this file. |
| `FbaOptFileLineFormat` | `FbaOptOutputFile.getFbaOptFileLineFormat()`             Returns the FBA optimization output file line format. |
| `FbaOptFileLineFormat` | `CofoofSource.getFbaOptFileLineFormat()`             Returns the FBA optimization file line format used to write data strings to files produced by this source. |

Constructors in sbrt.shell.io.fba with parameters of type FbaOptFileLineFormat

`CofoofSource(java.util.Iterator<java.lang.String> fileNameSource,
FileFormat fileFormat,
java.util.Set<java.lang.String> columnHeaders,
FbaOptFileLineFormat fbaFormat)`
  
          Constructs a new source of column-oriented FBA optimization output files.

`CofoofSource(java.util.Iterator<java.lang.String> fileNameSource,
java.lang.String fileNameSourceName,
FileFormat fileFormat,
java.util.Set<java.lang.String> columnHeaders,
FbaOptFileLineFormat fbaFormat)`
  
          Constructs a new source of column-oriented FBA optimization output files.

`ColumnOrFbaOptOutputFile(java.lang.String fileName,
FileFormat fileFormat,
java.util.Set<java.lang.String> columnHeaders,
FbaOptFileLineFormat fbaFormat)`
  
          Constructs a new column-oriented FBA optimization output file.

`RofoofSource(java.util.Iterator<java.lang.String> fileNameSource,
FileFormat fileFormat,
java.util.Set<java.lang.String> rowHeaders,
FbaOptFileLineFormat fbaFormat)`
  
          Constructs a new source of row-oriented
FBA optimization output files.

`RofoofSource(java.util.Iterator<java.lang.String> fileNameSource,
java.lang.String fileNameSourceName,
FileFormat fileFormat,
java.util.Set<java.lang.String> rowHeaders,
FbaOptFileLineFormat fbaFormat)`
  
          Constructs a new source of row-oriented
FBA optimization output files.

`RowOrFbaOptOutputFile(java.lang.String fileName,
FileFormat fileFormat,
FbaOptFileLineFormat fbaFormat)`
  
          Constructs a new row-oriented FBA optimization output file.

`RowOrFbaOptOutputFile(java.lang.String fileName,
FileFormat fileFormat,
java.util.Set<java.lang.String> rowHeaders,
FbaOptFileLineFormat fbaFormat)`
  
          Constructs a new row-oriented FBA optimization output file.

`SerFbaOptOutputFile(java.lang.String fileName,
FileFormat fileFormat,
java.util.Set<java.lang.String> filterKeys,
FbaOptFileLineFormat fbaFormat)`
  
          Constructs a new serialized FBA optimization output file.

`SfoofSource(java.util.Iterator<java.lang.String> fileNameSource,
FileFormat fileFormat,
java.util.Set<java.lang.String> filterKeys,
FbaOptFileLineFormat fbaFormat)`
  
          Constructs a new source of serialized
FBA optimization output files.

`SfoofSource(java.util.Iterator<java.lang.String> fileNameSource,
java.lang.String fileNameSourceName,
FileFormat fileFormat,
java.util.Set<java.lang.String> filterKeys,
FbaOptFileLineFormat fbaFormat)`
  
          Constructs a new source of serialized
FBA optimization output files.

Uses of FbaOptFileLineFormat in sbrt.shell.text.fba

| Classes in sbrt.shell.text.fba that implement FbaOptFileLineFormat | |
| --- | --- |
| `class` | `FbaOptFileLineFormatV1`             This class is a concrete implementation of `FbaOptFileLineFormat`. |

---


|  |  |  |
| --- | --- | --- |
| **Overview** \| **Package** \| **Class** \| **Use** \| **Tree** \| **Deprecated** \| **Index** \| **Help** | |  |
| PREV   NEXT | **FRAMES**    **NO FRAMES**     **All Classes** |  |


---
